# Systemic paracoccidioidomycosis in a patient with enzymatic myeloperoxidase deficiency and acute lymphoblastic leukemia: case report

**DOI:** 10.1186/s42269-022-00973-z

**Published:** 2022-12-28

**Authors:** Sebastián Hoyos, Cristián Camilo Agudelo, Diana Mercedes Lozano, Luis Eduardo Buitrago

**Affiliations:** 1 Stem Cell Transplant Unit, Clínica Somer, Rionegro, Antioquia Colombia; 2 Internal Medicine Resident, Universidad de Antioquia, Medellín, Antioquia Colombia; 3 Pathology Laboratory, Clínica Somer, Rionegro, Antioquia Colombia; 4 Stem Cell Transplant Unit, Hospital Pablo Tobón Uribe, Medellín, Antioquia Colombia

**Keywords:** Case report, Myeloperoxidase deficiency, Paracoccidioidomycosis, Acute lymphoblastic leukemia lymphoblastic, Enzymatic deficiency

## Abstract

**Background:**

Paracoccidioidomycosis is a systemic fungal infection that is potentially fatal, and the most prevalent of its kind in Latin America. The predisposition to infection appears to be related to abnormalities in cellular immunity, given its low prevalence in endemic regions. The role of myeloperoxidase deficiency has not been elucidated.

**Case presentation:**

We present a case of 48-year-old female patient with acute lymphoblastic leukemia, stem cell transplant candidate, who developed a fever with lymphadenopathy and lung nodules, consistent with paracoccidioidomycosis infection, in whom a myeloperoxidase deficiency was later discovered. The treatment of the hematologic malignancy had a good impact solving the enzymatic deficiency and antifungal therapy achieve controlling the infection.

**Conclusions:**

This case lays out the possible association between acute leukemia, an alteration in neutrophil function (needed to fight fungal infections) and an infection due to *Paracoccidioides brasiliensis*.

## Background

Myeloperoxidase (MPO) deficiency is a type of condition which affects the antimicrobial capacity of phagocytes. It can occur due to a hereditary condition (autosomic recessive inheritance) or less commonly, acquired through somatic mutations (Pahwa et al. 2022).

MPO composes the azurophilic granules of neutrophiles, but it is also present in monocytes and macrophages. This enzyme is released to the phagolysosome as adjuvant in the respiratory burst, using hydrogen peroxide and chloride to produce hypochloric acid, which favors the microbicidal effect within the phagocyte (Davies 2021).

Paracoccidioidomycosis is a systemic mycosis with an acute course, caused by the thermally dimorphic fungal complex *Paracoccidioides brasiliensis,* which is present in wet zones of Latin America, where it is endemic. It is estimated that 50% of the residents of endemic areas have had contact with this fungus. Most of the cases are found in Brazil, but the Endemic is also high in Colombia, Venezuela and northeastern Argentina (Arenas Guzmán et al. 2020). The reported fatality in endemic countries oscillates between 6.1 and 7.6% (Restrepo-Moreno et al. 2021).

The infection starts with the inhalation of the fungus which causes inflammation in the lungs. Later, it spreads to the skin, mucous membranes and lymph nodes, but also affects the viscera, especially the stomach, intestine, spleen and adrenal glands. The immune system via lymphocyte T helper—1 stimulation, tends to form granulomas to control the infection, from which latent yeasts can reactivate in immunosuppression states (Trujillo 2020). 

The pathogenesis of this mycosis is dependent on the virulence factor of the fungus, environmental conditions, and the effectiveness of the host immune response, in which the innate and acquired immune system are key figures. The former plays a leading part, shown in in vitro studies that exhibit the role of neutrophils as an immunologic barrier, and in histological findings from *P. brasiliensis* granulomas, where polymorphonuclear neutrophils are the predominant cell (Restrepo-Moreno et al. 2021; Dias et al. 2008).

For this case, we present a patient with a recent diagnosis of early T cell precursor acute lymphoblastic leukemia (ALL), whom was considered to start treatment based on the Spanish PETHEMA (*Programa Español de Tratamientos en Hematología*) chemotherapy protocol in whom MPO deficiency was later identified, in relation to a systemic fungal infection due to *Paracoccidioides brasiliensis.* In the current available literature, the role of immunodeficiency that our patient presented has not been elucidated. It should be noted that even though said fungus is not an opportunistic pathogen, it can cause disease when one or various host defense mechanisms are altered.

## Case presentation

A 48-year-old Colombian female, Caucasian, without relevant personal or familiar medical history, who had been diagnosed with early T cell precursor ALL, with high-risk criteria due to her age and malignancy subtype who was initially treated with the PETHEMA ALL AR 2011 protocol, and with prophylactic intrathecal chemotherapy, starting in January of 2022.

**Fig. 1:**
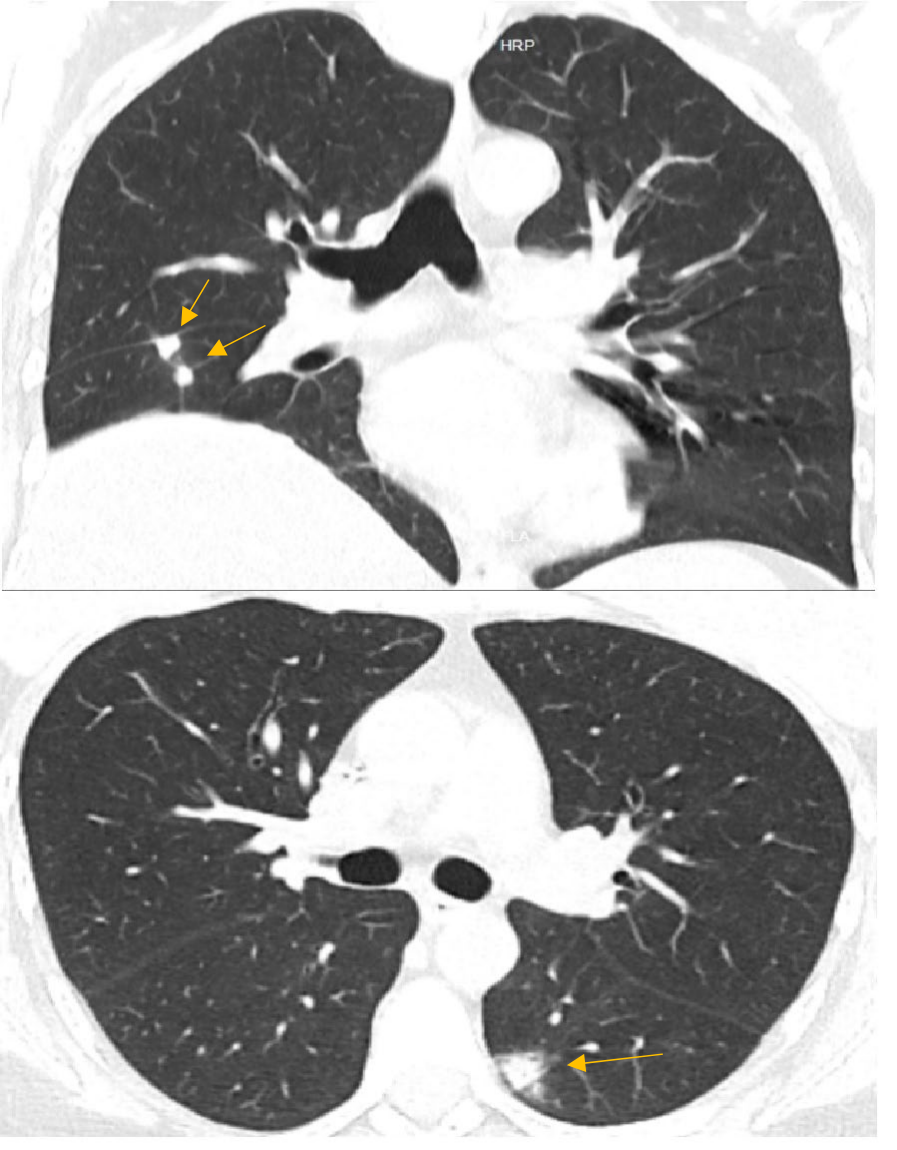
High resolution chest computed tomography showing bilateral solid nodules with ground glass halo (arrows). Source: Authors

During her initial hospital stay, she presented with fever, along with chest computed tomography findings of ground-glass nodules in the lungs (Fig. 1) and was started on Voriconazole, due to a high suspicion of invasive pulmonary aspergillosis infection.

The assessment of serum galactomannan was negative. Subsequently she exhibited a rise in aminotransferase levels over 6 times the upper limit of normal, along with conjugated hyperbilirubinemia. After performing magnetic resonance imaging, autoantibody tests, serologies for hepatotropic viruses and a liver biopsy, which ruled out fungal infection or infiltration due to her hematologic malignancy, it was concluded that she was experiencing drug toxicity due to her antifungal treatment. Therapy was changed to Isavuconazole which was administered for about 2 weeks.

Despite these efforts, the patient had recurrent fever episodes and lung nodules persisted. A bronchoscopy with bronchoalveolar lavage was performed, but no relevant pathogens where isolated in the samples. SARS-COV-2, Tuberculosis molecular assays, Galactomannan and fungal cultures were negative.

**Fig. 2:**
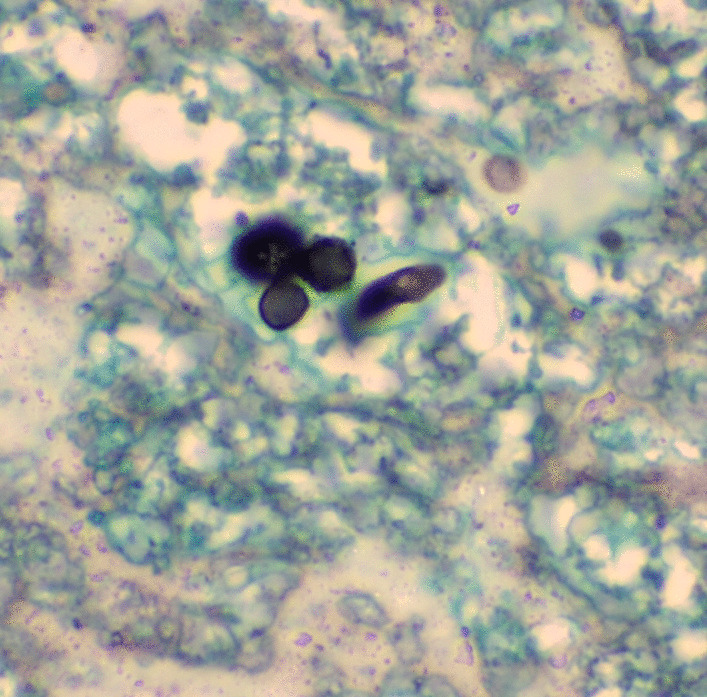
Round, thick-walled conidia, with multibudding yeast cells (“Mickey Mouse ears”), compatible with *Paracoccidioides brasiliensis.* Grocott’s silver methenamine stain, 40 ×. Source: Authors

**Fig. 3:**
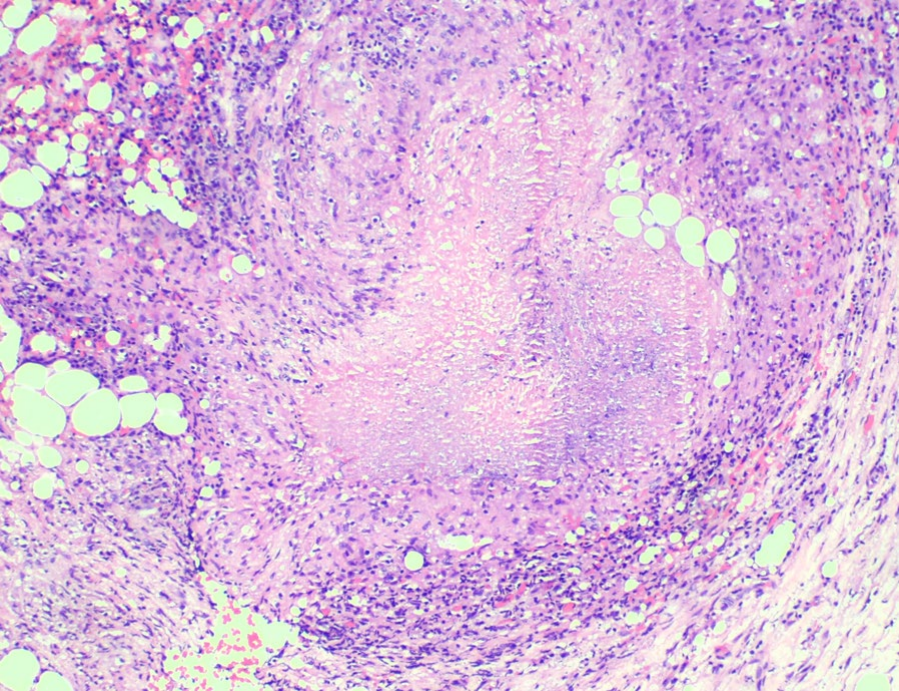
Epithelioid histiocyte aggregate that constitute a granuloma with central caseating necrosis. H & E, 10 × Source: Authors

Multiple cervical lymphadenopathies of several centimeters in diameter where later detected. A biopsy was performed to rule out possible T cell lymphoblastic leukemia or a systemic mycosis, which had not been identified up to that moment. Ziehl–Neelsen stain and polymerase chain reaction assays for tuberculosis were negative, but the histopathologic study exhibited chronic necrotizing granulomatous inflammation, with large areas of liquefactive necrosis, containing *Paracoccidioides brasiliensis* yeasts (Figs. 2, 3).

Following this, infectious disease specialists determined treatment with Liposomal Amphotericin B, and after 1 week of treatment fever seized, cervical lymph node size gradually reduced, and 3 weeks later, lung nodules disappeared.

**Fig. 4:**
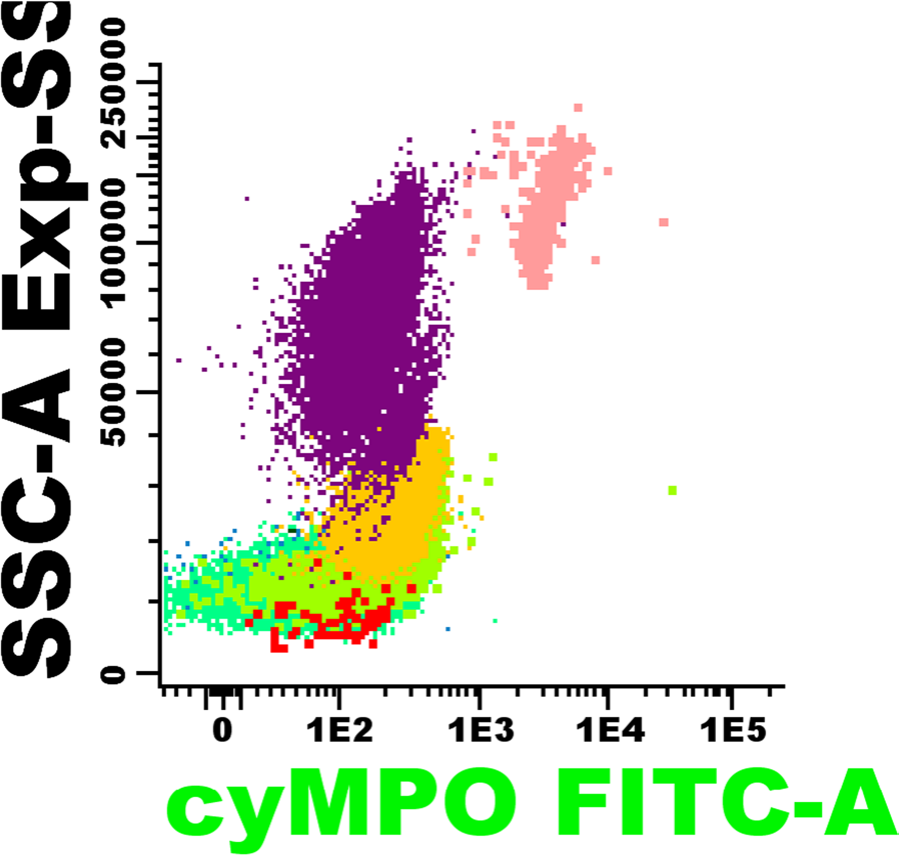
Bone marrow flow cytometry reveals neutrophils (purple) and monocytes (yellow) lacking expression of cytoplasmic Myeloperoxidase. Eosinophils are depicted in pink, mature T lymphocytes in green (seafome green like), dendritic cells in green (lime green like) and residual T lymphoblast in red. Abbreviations: cyMPO, cytoplasmic Myeloperoxidase; FITC, fluorescein isothiocyanate; SSC-A, side scatter. Source: Authors

Regarding ALL, the treatment was resumed and after completion of the chemotherapy regimen, flow cytometry was performed in a bone marrow sample, which reported 0.4% of lymphoblasts, and MPO deficiency, depicted in Fig. 4, where flow cytometry shows different white cell populations as polymorphonuclear neutrophils and monocytes negative for MPO fluorescence, as an incidental finding as shown. This discovery correlates with the presence of Large Unstained Cells (LUC)/false neutropenia in the complete blood count, with a normal peripheral blood smear.

As part of the treatment of the ALL a haploidentical allogenic stem cell transplant process was later initiated. During this process, she had multiple infectious complications, intestinal bleeding, and cholestasis, which lead to the discontinuation of liposomal amphotericin B. Antifungal treatment was stopped for 2 weeks and was later restarted using Trimethoprim/Sulfamethoxazole as indicated by the infectious diseases specialist. By that time, a control chest computed tomography did not report any lung nodules.

During her bone marrow transplant recovery and engraftment, findings consistent with resolution of the MPO deficiency were noted (increase in the number of positive MPO neutrophils in the blood count). Finally, after making a complete recovery, she was discharged, and treatment with Trimethoprim/Sulfamethoxazole was continued for 12 additional months. Two weeks after discharge, a new blood count was obtained, which showed MPO positive neutrophils and stability of the graft.

## Discussion

Our patient presented with a hematologic malignancy and was a candidate for stem cell transplant. During the treatment of her condition, MPO deficiency and an infection due to *Paracoccidioides brasiliensis,* were documented.

As described earlier, MPO has a fundamental role in the phagolysosomal complex as a microbicide, and its acquired or hereditary deficit, constitutes one of the most frequent immunodeficiencies, being directly associated with *Candida *spp*.,* infections (Davies 2021), yet its relation to another systemic mycosis has been poorly studied.

Previous studies have suggested a neutrophil disorder in some point of phagocyting, as an implicated factor in the increased susceptibility for acquiring a Paracoccidial disease (Dias et al. 2008; Domingues-Ferreira et al. 2017; Goihman-Yahr et al. 2009). It has even been linked with an incapacity to destroy the fungus, due to a deficit in free radical production, which are central to the respiratory burst.

Regarding this case, a single report in the literature was found of a patient who presented with systemic infections due to *P. brasiliensis* and *M. tuberculosis,* and given his clinical characteristics, was submitted to an immunodeficiency panel, which revealed an MPO deficiency as the only potential predisposing factor (Domingues-Ferreira et al. 2017).

Additionally, a correlation between acute myeloid leukemia and the development of MPO deficiency has been reported, associated with a disfunction in the Golgi apparatus of clonal cells (Catovsky et al. 2009). Given that early T cell blasts are immature cells that share certain characteristics with blasts from other cell lines, it is possible that a relationship may exist between this subtype of leukemia and an MPO deficiency, for which more studies are required.

The PETHEMA chemotherapy protocol includes drugs such as Vincristine, Daunorubicin and L-asparaginase, which have the potential to cause myelosuppression and leukocyte alterations (Guía 2013). The latter alters innate and adaptative immune mechanisms and renders the patient more susceptible to infections by different agents.

It is important to emphasize that one of the differential diagnosis of this case is tuberculosis infection as the patient lives in an endemic country, had conditions that altered her immune response and the clinical picture with prolonged fever, lung involvement and lymphadenopathies was compatible, but Ziehl–Neelsen stains, cultures and polymerase chain reaction assays in broncoalveolar lavage and her neck adenopathy biopsy were all negative.

Another differential diagnosis was sarcoidosis, but typical findings of bilateral hilar adenopathy, mediastinal enlarged lymph nodes were absent, and the granuloma found in the lymph node biopsy was caseating making it a less likely diagnosis.

Probably, in the case of our patient, the convergence of these factors was pivotal to the development of the infection, including the hematologic malignancy with its chemotherapy treatment, and finally the MPO deficiency, which based on what was previously described, could be one of the most important predisposing factors and could have an association with the hematologic malignancy. It is worth to highlight the complexity of this case, given that the patient was subjected to an allogenic stem cell transplant, without finishing the antifungal treatment regimen. After this, it was noted that automated blood counts started showing normal neutrophil numbers, and the system never again reported LUC.

Considering current evidence, new experimental studies, where neutrophil’s MPO activity is quantified as they are exposed to *P. brasiliensis* samples, could explore the theory that is proposed in this article.

## Conclusions

Based on our knowledge, this is the first case exposing a possible association between lymphoblastic T cell leukemia, MPO deficiency and a systemic infection due to *P. brasiliensis.* Although it is not considered an opportunistic mycosis, the convergence of these immunosuppressive factors could have facilitated the systemic compromise in this patient.

## Data Availability

Data sharing is not applicable to this article as no datasets were generated or analyzed during the current study.

## Abbreviations

MPO: Myeloperoxidase
ALL: Acute lymphoblastic leukemia
LUC: Large unstained cells
